# Causation and familial confounding as explanations for the associations of polygenic risk scores with breast cancer: Evidence from innovative ICE FALCON and ICE CRISTAL analyses

**DOI:** 10.1002/gepi.22556

**Published:** 2024-03-12

**Authors:** Shuai Li, Gillian S. Dite, Robert J. MacInnis, Minh Bui, Tuong L. Nguyen, Vivienne F. C. Esser, Zhoufeng Ye, James G. Dowty, Enes Makalic, Joohon Sung, Graham G. Giles, Melissa C. Southey, John L. Hopper

**Affiliations:** ^1^ Centre for Epidemiology and Biostatistics, Melbourne School of Population and Global Health The University of Melbourne Carlton Victoria Australia; ^2^ Centre for Cancer Genetic Epidemiology, Department of Public Health and Primary Care University of Cambridge Cambridge UK; ^3^ Precision Medicine, School of Clinical Sciences at Monash Health Monash University Clayton Victoria Australia; ^4^ Murdoch Children's Research Institute Royal Children's Hospital Parkville Victoria Australia; ^5^ Genetic Technologies Ltd. Fitzroy Victoria Australia; ^6^ Cancer Epidemiology Division Cancer Council Victoria Melbourne Victoria Australia; ^7^ Division of Genome and Health Big Data, Department of Public Health Sciences, Graduate School of Public Health Seoul National University Seoul Korea; ^8^ Genomic Medicine Institute Seoul National University Euigwahakgwan #402, Seoul National University College of Medicine, 103, Daehak‐ro, Jongno‐gu Seoul South Korea; ^9^ Institute of Health and Environment Seoul National University 1st GwanakRo Seoul South Korea; ^10^ Department of Clinical Pathology The University of Melbourne Parkville Victoria Australia

**Keywords:** breast cancer, causation, familial confounding, family data, ICE CRISTAL, ICE FALCON, siblings

## Abstract

A polygenic risk score (PRS) combines the associations of multiple genetic variants that could be due to direct causal effects, indirect genetic effects, or other sources of familial confounding. We have developed new approaches to assess evidence for and against causation by using family data for pairs of relatives (Inference about Causation from Examination of FAmiliaL CONfounding [ICE FALCON]) or measures of family history (Inference about Causation from Examining Changes in Regression coefficients and Innovative STatistical AnaLyses [ICE CRISTAL]). Inference is made from the changes in regression coefficients of relatives' PRSs or PRS and family history before and after adjusting for each other. We applied these approaches to two breast cancer PRSs and multiple studies and found that (a) for breast cancer diagnosed at a young age, for example, <50 years, there was no evidence that the PRSs were causal, while (b) for breast cancer diagnosed at later ages, there was consistent evidence for causation explaining increasing amounts of the PRS‐disease association. The genetic variants in the PRS might be in linkage disequilibrium with truly causal variants and not causal themselves. These PRSs cause minimal heritability of breast cancer at younger ages. There is also evidence for nongenetic factors shared by first‐degree relatives that explain breast cancer familial aggregation. Familial associations are not necessarily due to genes, and genetic associations are not necessarily causal.

## 1 INTRODUCTION

A polygenic risk score (PRS) combines the associations of multiple genetic variants with a given trait. Although a PRS—like any other risk factor—can be used to predict the likelihood of having the trait now or in the future, is there a causal aspect to this association that could be used to inform prevention and aetiological research? Use in the literature of phrases like ‘confer risk’ or ‘convey risk’ imply a causal interpretation, but is this just the writer who is doing the conferring (labelling) and conveying (putting ideas into the reader's mind) rather than the implicated genes and/or genetic variants themselves being biologically relevant to the disease in question?

Associations can always be due to confounding, whereby there is an external factor (confounder) that influences both the exposure and the outcome. Genetic associations, and therefore genetic predictions, could therefore be due to familial confounding in which the external factor is also correlated to some extent in relatives. For example, a PRS‐trait association does not imply that all or even any of the measured variants are causal because variants can be ‘tagged’ by truly causal variants. That is, on a population basis a truly causal genetic variant could be a confounder for the trait association of a measured genetic variant included in the PRS.

It is also possible for a PRS‐trait association to be due at least in part to other sources of familial confounding. Some possibilities include (i) population stratification, in which ancestry is correlated with both phenotypes and genotypes due to geographic or regional differences in allele frequency relating to the trait of interest; (ii) assortative as distinct from random mating, whereby people mate with those of more similar phenotypes for whatever reason resulting in parents being more similar for the genetic aspects underlying those phenotypes than by chance and (iii) indirect genetic effects in which the expression of a parental genotype on the parental phenotype directly affects the offspring phenotype (Brumpton et al., 2020). There could be other reasons as well.

For breast cancer, both a 77‐single‐nucleotide polymorphism (SNP) PRS (Mavaddat et al., 2015) and more recently a 313‐SNP PRS (Mavaddat et al., 2019) have been shown to predict disease risk with the odds ratio per cross‐sectional standard deviation of the PRS (odds ratio [OR]) estimated to be 1.49 (95% confidence interval [CI]: 1.44, 1.56) and 1.61 (95% CI: 1.57, 1.65), respectively. The 77‐SNP PRS was created using a significance threshold of *p* < 5 × 10^−8^ and a sample size of 33,673 cases and 33,381 controls, while the 313‐SNP was created using *p* < 10^−5^ and a sample size 94,075 cases and 75,017 controls. Perhaps the truly causal variants are more likely to be captured by PRSs created from studies that use more stringent thresholds, or apply to different subtypes of disease related to age at diagnosis, hormone‐receptor status and so on. To the best of our knowledge, the causal aspects of the breast cancer PRSs have not been addressed.

Mendelian randomisation, which uses genetic variants associated with exposures as presumed instrumental variables to assess causal evidence, cannot be used to investigate the causality of a PRS. This is because the PRS is the exposure underlying this research question and is genetic itself—there are no genetic instrumental variables for a PRS.

Family designs have historically been used to investigate the causes of variation (i.e. familial and nonfamilial associations) in measured nonbinary traits. They address the strengths of familial associations, not necessarily the issue of causes per se.

We have developed innovative methods to assess evidence for and against causation between an exposure and an outcome that apply regression analyses to family data. The first method is called Inference about Causation from Examination of FAmiliaL CONfounding (ICE FALCON) (Li et al., 2020), which uses data from pairs of relatives, such as twins or siblings. The exposure of the relative is used as a measured proxy instrumental variable for assessing causal evidence but without the assumption that there are no other causal pathways from the relative's exposure to the outcome. Importantly, inference using ICE FALCON does not come from fitting one model, but instead from fitting multiple models and studying the changes in pairs of regression coefficients. ICE FALCON analysis has provided causal evidence for numerous exposures and outcomes (Bui et al., 2013, 2020; Dite et al., 2008; Dongmeng et al., 2022; Hong et al., 2023; Hopper et al., 2012; Li et al., 2018, 2019, 2020; Nissen et al., 2023; Stone et al., 2012; Tian et al., 2023; Wu et al., 2022; Zheng et al., 2022).

A more recent method is the Inference about Causation from Examining Changes in Regression coefficients and Innovative STatistical AnaLyses (ICE CRISTAL) (Li & Hopper, 2023), which in effect extends ICE FALCON by using the relative's outcome (i.e. a person's family history, however defined) as the proxy instrumental variable. ICE CRISTAL is therefore more generally applicable as it does not rely on the exposure of the relative to be measured and can use data for unrelated individuals. ICE CRISTAL previously provided evidence consistent with mammographic density having a causal effect on breast cancer risk (Martin et al., 2010), although this insight was not made at the time. More details about these methods can be found in the Methods section.

In this study, we aimed to investigate the evidence for causality of the breast cancer PRSs using the ICE FALCON and ICE CRISTAL methods. We investigated the causal effects overall, and by age at diagnosis, using data from three cohorts and published data.

## 2 METHODS

### 2.1 ICE FALCON analysis

**Figure 1:**
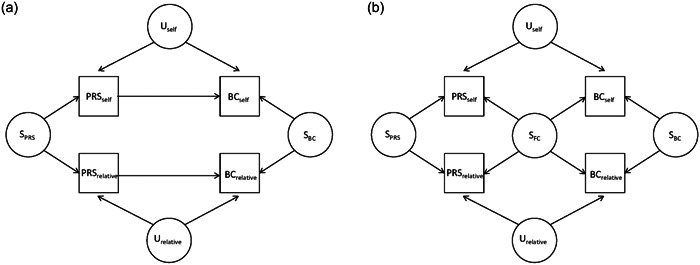
Possible causal diagrams between PRS and breast cancer for pairs of relatives: (a) causation and (b) familial confounding. PRS represents a polygenic risk score and BC represents breast cancer. S denotes the combinations of all unmeasured factors (genetic and/or nongenetic) that affect both relatives: S_PRS_ represents those causing the PRS only, S_BC_ represents those causing breast cancer only, and S_FC_ represents those causing both the PRS and breast cancer (i.e., familial confounders). U denotes all the unmeasured individual‐specific confounders between PRS and breast cancer that are not shared by relatives. For the purposes of explanation, ‘self’ refers to a woman and ‘relative’ refers to the woman's relative but recognise that these labels can be swapped and both relatives within a pair are included in the analyses.

Figure 1 shows two possible causal diagrams between PRS and breast cancer for pairs of relatives: (1) PRS has a causal effect on breast cancer (Figure 1a), and (2) the association between PRS and breast cancer is due to familial confounding, that is, there is no causation (Figure 1b). ICE FALCON fits three models:

1. *E* (BC_self_) = *α* + *β*_self_PRS_self_, which estimates the marginal association between a woman's breast cancer and her own PRS (*β*_self_).
2. *E* (BC_self_) = *α *+ *β*_relative_PRS_relative_, which estimates the marginal association between a woman's breast cancer and her relative's PRS (*β*_relative_).
3. *E* (BC_self_) = *α* + *β*′_self_PRS_self_ + *β*′_relative_PRS_relative_, which estimates the conditional association between a woman's breast cancer with her own PRS given her relative's PRS (*β*′_self_), and the conditional association between a woman's breast cancer with her relative's PRS given her own PRS (*β*′_relative_).

Observing that PRS_self_ association does not change after conditioning on PRS_relative_ (i.e. *β*′_self_ − *β*_self_ ~ 0) but that PRS_relative_ association decreases to the null after conditioning on PRS_self_ (i.e. *β*′_relative_ ~ 0 and *β*′_relative_ − *β*_relative_ < 0) is consistent with PRS having a causal effect on breast cancer (Figure 1a). Observing that both PRS_self_ association and PRS_relative_ association decrease to the same extent after conditioning on each other (i.e. *β*′_self_ − *β*_self_ ~ *β*′_relative_ − *β*_relative_) is consistent with there being familial confounding (Figure 1b). For a scenario in which there is a mixture of causation and familial confounding, the results are expected to be a mixture of the results of the two specific scenarios. That is, asymmetry in the changes in regression coefficients is indicative of causation, even in a setting where there is also familial confounding. More details about ICE FALCON can be found in Li et al. (2020).

### 2.2 Application of ICE FALCON

We applied ICE FALCON to genotyped sister pairs from the UK Biobank. As in Brumpton et al. (2020), genetic data were used to identify full siblings among UK Biobank participants, resulting in 41,496 siblings in 20,136 families. We selected siblings who were female and unaffected by breast cancer at enrolment, and families with at least two sisters, resulting in 13,696 sisters in 6847 families. For families with more than two sisters, we generated all possible sister pairs. As a result, a total of 7410 sister pairs were included in the analysis. Breast cancer diagnosis after enrolment (i.e. incident cancers) was determined using self‐reported and linked cancer registry data (ICD9 code 174 or ICD10 code C50); only the first incident invasive breast cancer diagnosis was considered. The affected women had an average age at diagnosis of 64 years. The 313‐SNP PRS was calculated using published estimates of the OR per effect allele and effect allele frequency (Mavaddat et al., 2019) and scaled to have a population average risk of 1; see Spaeth et al. (2023) for more details. ICE FALCON analysis was conducted for the whole sample, and by age groups (<50 years, 50–60 years, ≥60 years). Both sisters of a pair were included in the analysis, and generalised estimating equation was used to account for the relatedness between sisters. Age was included in the models as a covariate. Association estimates were reported as the Odds ratio PER Adjusted standard deviation (OPERA) (Hopper, 2015). The causal effect of a PRS was calculated as log (OR) = ([*β*_relative_ − *β*′_relative_] − [*β*_self_ − *β*′_self_])/*ρ*, where *ρ* is the correlation in the PRS between sister pairs. Standard errors for the changes in regression coefficient estimates between models and for the causal effect were estimated using bootstrapping, as in Li et al. (2020).

### 2.3 ICE CRISTAL analysis

ICE CRISTAL extends ICE FALCON by replacing PRS_relative_ with BC_relative_ in Models 2 and 3 above. BC_relative_ is a component of a woman's breast cancer family history; therefore, BC_relative_ can be further replaced with any measure of family history and then data for unrelated individuals can be used. That is, ICE CRISTAL estimates the marginal associations of a woman's breast cancer diagnosis with her own PRS, and with her family history, and then the conditional associations from fitting her PRS and her family history together, and finally considers the changes in the pairs of association estimates. Observing that the PRS association does not change after conditioning on family history, but that the family history association decreases after conditioning on PRS, is consistent with PRS having a causal effect on breast cancer. Familial confounding is consistent with both the PRS association and the family history association decreasing. Combinations of causation and familial confounding can occur, with the results are expected to a mixture of those arising from causation and those due to familial confounding.

### 2.4 Applications of ICE CRISTAL analysis

We applied ICE CRISTAL to three studies. The first is a case‐control study using the Australian Breast Cancer Family Registry (ABCFR), which recruited population‐based breast cancer case families and age‐matched control families and was designed to be enriched for breast cancer diagnosed at younger ages (Hopper et al., 1994, 1999; McCredie et al., 1998). A total of 750 breast cancer cases and 405 controls younger than 50 years old at diagnosis and recruitment, respectively, were included in this analysis. The average age at diagnosis of cases was 38 years. The 77‐SNP PRS was calculated using published estimates of the OR per effect allele and effect allele frequency (Mavaddat et al., 2015) and scaled to have a population average risk of 1, using the same method as above when applying ICE FALCON to the UK Biobank. Family history was used to calculate a familial risk score defined as the 5‐year absolute risk of invasive breast cancer using the Breast and Ovarian Analysis of Disease Incidence and Carrier Estimation Algorithm (BOADICEA) (Antoniou et al., 2004, 2008). More details about the sampling, PRS and familial risk score calculations can be found in Dite et al. (2016). Logistic regression was used to fit the three ICE CRISTAL models, with age as a covariate. Associations were reported as the OPERAs of the PRS and familial risk score.

The second is a cohort study using the UK Biobank based on 200,195 women who were breast cancer free at enrolment. We considered the first invasive breast cancer diagnosis within 5 years of follow‐up since baseline assessment. The sample included 197,057 unaffected women who had an average age of 57 years at baseline assessment, and 3138 affected women who had an average age of 58 years at baseline assessment and an average age at diagnosis of 61 years. The 77‐SNP PRS and 313‐SNP PRS were calculated as described above. Breast cancer family history in first‐degree relatives (mother and/or sisters) was self‐reported by the women at baseline assessment and analysed as a three‐level categorical variable (no family history, one relative affected, two relatives affected). More details about the sampling and PRS calculation can be found in Spaeth et al. (2023). We used Cox regression to fit the three ICE CRISTAL models and estimated the hazard ratios per adjusted standard deviation of PRS and for family history, respectively.

The third is a case‐cohort study nested in the Melbourne Collaborative Cohort Study (MCCS) of 24,469 women from Melbourne, Australia, aged between 27 and 76 years (99% were 40–69 years) at recruitment (Milne et al., 2017). The sample included 408 women diagnosed with the first invasive breast cancer within 10 years of follow‐up and a random sample of 2783 women attending at baseline, of whom 93 were cases. The average age at diagnosis of cases was 68 years. Two 10‐year absolute risk scores based on the 313‐SNP PRS and family history were calculated using the BOADICEA risk model (Version 5) (Lee et al., 2019), respectively. More details about the sampling and risk score calculations can be found in Li et al. (2021). We used Cox regression to fit the three ICE CRISTAL models and estimated the hazard ratios per 10‐year risk for the two risk scores.

### 2.5 Further applications of ICE CRISTAL

We also examined the published data from essentially ICE CRISTAL analyses in the Breast Cancer Association Consortium (BCAC) and FinnGen. In the BCAC studies (Mavaddat et al., 2015, 2019), PRSs included the 77‐SNP PRS and 313‐SNP PRS, and family history was defined by having breast cancer in at least one first‐degree relative. The 77‐SNP PRS analysis involved 21,865 breast cancer cases and 15,830 controls (Mavaddat et al., 2015), and the 313‐SNP PRS analysis involved 6787 ER‐positive cancer cases and 17,351 controls and 4440 ER‐negative cancer cases and 13,132 controls (Mavaddat et al., 2019).

In the FinnGen study (Mars et al., 2022), the PRS was genome‐wide and consisted of 1,079,089 SNPs. There were three measures of family history: (1) breast cancer in at least one first‐degree relative, (2) breast cancer in at least one second‐degree relative and (3) breast cancer as the parental cause of death. The sample sizes were 886 cases and 14,395 controls, 881 cases and 18,092 controls and 8733 cases and 124,920 controls for the three measures of family history, respectively.

## 3 RESULTS

### 3.1 ICE FALCON results

Table 1 shows the results of ICE FALCON analyses applied to the UK Biobank sister pairs. Overall, a woman's breast cancer diagnosis was associated with her own PRS (OPERA = 1.51, 95% CI: 1.43, 1.60) and her sister's PRS (OPERA = 1.30, 95% CI: 1.22, 1.38). When the two PRSs were conditioned on each other (Model 3), the association with a woman's PRS remained unchanged, and the association with her sister's PRS attenuated 71% (*p* = 10^−16^) to be at best marginally significant (OPERA = 1.08, 95% CI: 0.99, 1.17; *p* = 0.07). These results are consistent with, overall, the PRS having a causal effect on breast cancer, and the OPERA of the causal effect was estimated to be 1.40 (95% CI: 1.21, 1.63), accounting for 81% of the association between PRS and breast cancer.

**Table 1:** ICE FALCON results from the UK Biobank.^a^

Coefficient	Model 1		Model 2		Model 3		Change		Causal effect OPERA (95% CI)	Proportion of association due to causation (%)
	OPERA (95% CI)	*p*	OPERA (95% CI)	*p*	OPERA (95% CI)	*p*	Estimate (SE)	*p*		
Age at diagnosis < 50 years (871 pairs)										
*β*_self_	1.34 (1.09, 1.63)	4.6 × 10^−3^			1.44 (1.14, 1.81)	1.8 × 10^−3^	0.073 (0.070)	0.30	—	—
*β*_sister_			0.91 (0.54, 1.55)	0.62	0.76 (0.48, 1.20)	0.24	−0.190 (0.109)	0.08		
Age at diagnosis 50–60 years (1589 pairs)										
*β*_self_	1.45 (1.27, 1.67)	1.0 × 10^−7^			1.41 (1.16, 1.71)	5.1 × 10^−4^	−0.031 (0.053)	0.56	1.32 (0.92, 2.39)	74.8
*β*_sister_			1.28 (1.06, 1.55)	0.01	1.09 (0.83, 1.44)	0.53	−0.157 (0.066)	0.02		
Age at diagnosis ≥ 60 years (2036 pairs)										
*β*_self_	1.58 (1.41, 1.78)	2.6 × 10^−14^			1.55 (1.34, 1.79)	5.2 × 10^−9^	−0.023 (0.032)	0.48	1.46 (1.13, 1.99)	82.9
*β*_sister_			1.29 (1.15, 1.45)	2.6 × 10^−5^	1.06 (0.91, 1.24)	0.46	−0.195 (0.041)	2.2 × 10^−6^		
Whole sample (7410 pairs)										
*β*_self_	1.51 (1.43, 1.60)	5.9 × 10^−49^			1.47 (1.37, 1.57)	3.3 × 10^−29^	−0.030 (0.017)	0.08	1.40 (1.21, 1.63)	81.3
*β*_sister_			1.30 (1.22, 1.38)	6.2 × 10^−17^	1.08 (0.99, 1.17)	0.07	−0.184 (0.002)	9.0 × 10^−17^		

Abbreviations: CI, confidence interval; ICE FALCON, Inference about Causation from Examination of FAmiliaL CONfounding; OPERA, Odds ratio PER Adjusted standard deviation; SE, standard error.

^a^ Notice that *β*_self_ is age‐dependent.

When broken down by age at diagnosis and therefore age of controls, similar results were found for pairs aged 50–60 years and pairs aged ≥60 years, with the causal effect accounting for 75% and 83% of the association, respectively. For ages <50 years, however, a woman's breast cancer diagnosis was associated with her own PRS but not significantly with her sister's PRS (OPERA = 0.91, 95% CI: 0.54, 1.55) despite the PRS being naturally correlated within sister pairs. There was no evidence that the two PRS associations changed after conditioning on each other. These results are inconsistent with the PRS having a causal effect on breast cancer before ages 50 years.

### 3.2 ICE CRISTAL results

Table 2 shows the results of ICE CRISTAL analyses of the three studies. In the ABCFR case‐control study in which all breast cancer cases were diagnosed <50 years (average diagnosis age: 38 years) and all controls were also <50 years old, both the 77‐SNP PRS association and family history association remained unchanged after conditioning on each other. These results are inconsistent with the 77‐SNP PRS having a causal effect on breast cancer diagnosed before age 50 years, or explaining family history as a risk factor for breast cancers diagnosed before age 50 years.

**Table 2:** ICE CRISTAL results from three cohorts and published data.

Predictors	Model 1	Model 2	Model 3	Change (%)
	OR or HR	OR or HR	OR or HR	
*Australian Breast Cancer Family Registry (age at diagnosis and age of controls < 50 years)*				
PRS (77 SNPs)	1.46		1.46	0
Family history (BOADICEA 5‐year risk)		1.80	1.80	0
*UK Biobank (Average age at diagnosis = 61 years)*				
Analysis 1				
PRS (77 SNPs)	1.62		1.61	1.2
Family history (one first‐degree relative affected)		1.58	1.51	9.9
Family history (two first‐degree relatives affected)		2.56	2.39	7.3
Analysis 2				
PRS (313 SNPs)	1.53		1.52	1.5
Family history (one first‐degree relative affected)		1.58	1.47	15.8
Family history (two first‐degree relatives affected)		2.56	2.31	10.9
*Melbourne Collaborative Cohort Study (average age at diagnosis = 68 years)*				
PRS (313 SNPs)	2.76		2.69	2.5
Family history (BOADICEA 10‐year risk)		1.84	1.20	70.0
*Breast Cancer Association Consortium*				
PRS (77 SNPs)	1.55		—	
Family history (first‐degree relatives)				
<40 years		2.90	2.76	4.6
40–60 years		1.88	1.72	14.1
≥60 years		1.63	1.53	13.0
Whole sample		1.81	1.68	12.6
*Breast Cancer Association Consortium*				
ER‐negative breast cancer				
PRS (313 SNPs)	1.44		—	
Family history (first‐degree relatives)		1.66	1.56	12.3
ER‐positive breast cancer				
PRS (313 SNPs)	1.67		—	
Family history (first‐degree relatives)		1.59	1.44	21.4
*FinnGen*				
Analysis 1				
PRS (1,079,089 SNPs)	1.71		1.66	5.5
Family history (first‐degree relatives)		2.36	1.99	19.9
Analysis 2				
PRS (1,079,089 SNPs)	1.84		1.83	0.9
Family history (second‐degree relatives)		1.47	1.38	16.4
Analysis 3				
PRS (1,079,089 SNPs)	1.76		1.76	0
Family history (parental cause of death)		2.02	1.74	21.2

Abbreviations: BOADICEA, Breast and Ovarian Analysis of Disease Incidence and Carrier Estimation Algorithm; HR, hazard ratio; ICE CRISTAL, Inference about Causation from Examining Changes in Regression coefficients and Innovative STatistical AnaLyses; OR, odds ratio; PRS, polygenic risk score; SNP, single‐nucleotide polymorphism.

In the UK Biobank study, where breast cancer cases had an average age at diagnosis of 61 years, both the 77‐SNP PRS association and the 313‐SNP PRS association remained unchanged after conditioning on family history, while the family history association attenuated 7%–10% after conditioning on the 77‐SNP PRS and 11%–16% after conditioning on the 313‐SNP PRS. These results are consistent with both the PRSs having causal effects on breast cancer diagnosed within 5 years of this older age range, with the 313‐SNP PRS having a larger effect.

Results from the MCCS study, in which the breast cancer cases had an average age at diagnosis of 68 years, were consistent with the 313‐SNP PRS having a causal effect on breast cancers diagnosed within 10 years of this older age range, as the PRS association remained unchanged after conditioning on family history, and the family history association attenuated 70% after conditioning on PRS.

Published results from the BCAC and FinnGen studies (Mars et al., 2022; Mavaddat et al., 2015, 2019) were also consistent with PRS having a causal effect (Table 2). In the BCAC study, the family history association attenuated 13% after conditioning on the 77‐SNP PRS. The attenuation was only 5% at ages <40 years, but greater at older ages. As for the 313‐SNP PRS, the family history associations with breast cancer subtypes attenuated after conditioning on it, with a greater attenuation for ER‐positive breast cancer. In the FinnGen study, family history associations attenuated 16%–21% after conditioning on the genome‐wide PRS, with the association for family history in a second‐degree relative having the smallest attenuation. The PRS association remained unchanged after conditioning on having a family history in a second‐degree relative or breast cancer as parental cause of death and attenuated 6% after conditioning on having a family history in a first‐degree relative.

## 4 DISCUSSION

Genetic epidemiology is about how genetic and nongenetic factors combine to influence health and disease, realising that familial associations might not necessarily due to genes, and that genetic associations might not necessarily be causal. Genetic epidemiology traditionally evolved from considering families, or at least sets of related individuals, as the unit of study and not isolated individuals alone. This study has taken genetic epidemiology back to its roots and used the experiment of opportunity arising from family studies to address causation, the Holy Grail of science, by applying the novel ICE FALCON and ICE CRISTAL concepts.

We found consistent evidence that the breast cancer PRSs could be picking up signals from breast cancer causes when the data were pooled over all ages at diagnosis, though not necessarily for breast cancers diagnosed at a young age such as before age 50 years. The causality might also be due to the genetic variants involved in the PRS being in linkage disequilibrium with truly causal variants and not necessarily being casual themselves. Evidence of causation came from both the ICE FALCON analyses of sibling pairs, and the individual‐specific ICE CRISTAL analyses of the family history association attenuation magnitude after conditioning on PRS, which increased with the number of variants included in the PRS: 77‐SNP PRS versus 313‐SNP PRS in the UK Biobank, and 313‐SNP PRS versus genome‐wide PRS in the FinnGen (Mars et al., 2022); a PRS consisting of more variants appears to be capturing the effects of more causal variants.

Leaving aside the paucity of evidence for the PRSs causing breast cancer diagnosed before age 50 years (see below), the causal effects being captured by the PRS are more evident and stronger for breast cancers diagnosed at older ages; ICE FALCON analyses found that the proportion of association due to causality increased with age and the ICE CRISTAL analyses found that the magnitude of family history attenuation after conditioning on PRS increased with age.

An important observation was that (1) a woman's breast cancer was not associated with her sister's PRS at ages <50 years in the UK Biobank study, (2) the family history association did not change after conditioning on PRS at ages <50 years in the ABCFR study and (3) the family history association only decreased 5% after conditioning on PRS at ages <40 years in the BCAC study. These observations imply that the PRSs explain minimal familial aggregation before menopause and at young ages, when the familial relative risks are greater, and are consistent with previous findings from segregation analyses (Li et al., 2022). This could be mostly due to the breast cancer familial risk, or familial variance (Hopper et al., 2023), decreasing with age (Collaborative Group on Hormonal Factors in Breast, 2001; Li et al., 2022; Wang et al., 2023) But the effect size of the within‐woman PRS association stays relatively stable with age (Li & Hopper, 2021; Li, 2021; Mavaddat et al., 2015, 2019), an intriguing observation given that risk associations should in general decrease with age as those at higher risk are preferentially ‘eliminated’ from older control groups. For colorectal and prostate cancer, the magnitudes of the PRS risk associations decrease substantially with age (Archambault et al., 2020; Li, 2021; Li & Hopper, 2021; Schaid et al., 2022; Thomas et al., 2020).

There is evidence that the PRS associations are confounded by nongenetic familial factors shared within nuclear families. This comes from observations reported by the FinnGen study that (1) the PRS association decreased 6% after conditioning on family history in first‐degree relatives, (2) there was no attenuation in the PRS association after conditioning on family history in second‐degree relatives and (3) the family history association decreased more after conditioning on a PRS when it was defined in terms of first‐degree rather than second‐degree relatives. This is further supported by the ICE FALCON analyses which found that there was a marginally statistically significant (*p* = 0.08) change in a woman's own PRS association after conditioning on her sister's PRS, and the sister's PRS association was marginally statistically significant (*p* = 0.07) in Model 3. As discussed in the Introduction, the familial confounding could be due to factors such as assortative mating, population stratification and indirect genetic effects (Brumpton et al., 2020).

There is evidence that there might be nongenetic factors contributing to breast cancer familial aggregation. Under the Variance of Age‐specific Log Incidence Decomposition (VALID) model (Hopper et al., 2023), the familial variance in breast cancer log(incidence) is given by *σ* ^2^ = log (FRR)/*r*, where FRR is the familial risk ratio for a given set of relatives and *r* is the correlation between those relatives in the familial causes. Assuming a model in which all of the breast cancer familial variance is due to genetic factors, the FRR for first‐degree relatives (in which case *r* = 0.5) in FinnGen predicts the familial variance to be 1.7, larger than the 1.5 based on the FRR for second‐degree relatives (in which case *r* = 0.25). The excess familial variance based on first‐degree relatives implies the existence of nongenetic familial factors. This is also suggested by analysis of twin pairs in the Nordic Twin Study which found evidence for nongenetic factors shared by twins, especially at younger ages (Hopper et al., 2023; Möller et al., 2016).

The study of Mars et al. (2022) using the FinnGen data investigated many other common diseases including other cancers, eye diseases, chronic kidney disease, and cardiometabolic, psychiatric, musculoskeletal, neurological, endocrine and allergic disorders. Consistent across the investigated diseases, they found that (1) there is evidence for nongenetic causes of disease familial aggregation, (2) the PRSs capture a substantial causal effect and (3) some of the nongenetic familial causes of diseases are confounded with PRS; see Li and Hopper (2023) for more discussion of the study implications.

Our study has demonstrated the unique value of the family‐based approaches of ICE FALCON and ICE CRISTAL in providing evidence for both causation and familial confounding. Unlike Mendelian randomisation, these two methods can be used to investigate the causal effect of PRS and are in general more powerful and flexible. Both methods use the measured relative's exposure or family history as a proxy for true instrumental variables that are not observed (i.e. familial causes that are truly specific to the exposure, not just assumed to be as in Mendelian randomisation). Not limited to measured ‘weak’ genetic variants as instrumental variables, the two methods can investigate all measured exposures, not just those for which there is substantial genetic knowledge as for Mendelian randomisation. The two methods can also make causal inference in the presence of familial confounding (in which the proxy instrumental variable is associated with the outcome through pathways not involving the exposure).

We have found here that the ICE FALCON and ICE CRISTAL methods both supported and complemented each other. The proxy instrumental variable is stronger in ICE FALCON than in ICE CRISTAL, so is more powerful because the relative's exposure is more correlated with a person's exposure than family history (i.e. the relative's outcome). On the other hand, ICE CRISTAL analysis is more widely applicable and can potentially be applied to more and larger sample sizes, given only a small proportion of studies collect data from relatives but most collect family history data. The two methods can be used to attempt to cross‐validate each other's findings using the same study, as we have shown.

In conclusion, the investigated breast cancer PRSs capture causal effects, at least for postmenopausal disease, and the causal effect is more evident and stronger at older ages. These current PRSs, however, cause minimal heritability of breast cancer at younger ages. There is also evidence for nongenetic factors shared by first‐degree relatives that also explain familial aggregation of breast cancer. Familial associations are not necessarily due to genes, and genetic associations are not necessarily causal.

## CONFLICT OF INTEREST STATEMENT

G. S. D. is an employee of Genetic Technologies Limited. The remaining authors declare no conflict of interest.

## Data Availability

The data that support the findings of this study are available from the corresponding author upon reasonable request.
